# Mechanisms of Long Non-Coding RNA in Breast Cancer

**DOI:** 10.3390/ijms24054538

**Published:** 2023-02-25

**Authors:** Bianca Giuliani, Chiara Tordonato, Francesco Nicassio

**Affiliations:** 1 Center for Genomic Science of IIT@SEMM, Istituto Italiano di Tecnologia (IIT), 20139 Milan, Italy; 2 IEO, European Institute of Oncology IRCCS, 20139 Milan, Italy; 3 Department of Oncology and Hemato-Oncology, Università degli Studi di Milano, 20122 Milan, Italy

**Keywords:** LncRNAs, cancer, breast, gene expression, MicroRNA, chromatin

## Abstract

The landscape of pervasive transcription in eukaryotic genomes has made space for the identification of thousands of transcripts that are difficult to frame in a specific functional category. A new class has been broadly named as long non-coding RNAs (lncRNAs) and shortly defined as transcripts that are longer than 200 nucleotides with no or limited coding potential. So far, about 19,000 lncRNAs genes have been annotated in the human genome (Gencode 41), nearly matching the number of protein-coding genes. A key scientific priority is the functional characterization of lncRNAs, a major challenge in molecular biology that has encouraged many high-throughput efforts. LncRNA studies have been stimulated by the enormous clinical potential that these molecules promise and have been based on the characterization of their expression and functional mechanisms. In this review, we illustrate some of these mechanisms as they have been pictured in the context of breast cancer.

## 1. Introduction

The next-generation sequencing era has strongly increased the number of annotated non-canonical transcripts, such as lncRNAs. There are several factors that make the current annotation of these non-coding transcripts challenging compared to protein-coding genes, since lncRNAs are (i) weakly conserved at the sequence levels during evolution, (ii) generally low-expressed and (iii) strongly context-dependent, meaning that their levels of expression can differ greatly between tissues or even different cell types within a tissue. So, it is not surprising that several databases such as LNCipedia, lncRNADisease 2.0, LncATLAS, LncRNAdb and Lnc2Cancer are not coherently concordant and need to be updated timely and be supported by biological validation [1,2,3,4,5]. The use of high-throughput data (including CAGE, RNA-seq and polyA site-seq) from available consortia such as ENCODE (https://www.encodeproject.org, accessed on 20 February 2023) or FANTOM (https://fantom.gsc.riken.jp, accessed on 20 February 2023) can be useful for characterizing the expression of a given candidate in a specific tissue/cell type. Multiple studies have highlighted the role of lncRNAs in diseases [6]. Expression studies comparing normal vs. cancer tissues have revealed many lncRNAs to be regulated in cancer, often with a very high cancer specificity. In addition, cancer pathways have been found to be regulated by intricated networks of coding and non-coding transcripts. As for coding transcripts, lncRNAs can have either tumor-suppressing or oncogenic functions [7].

**Figure 1:**
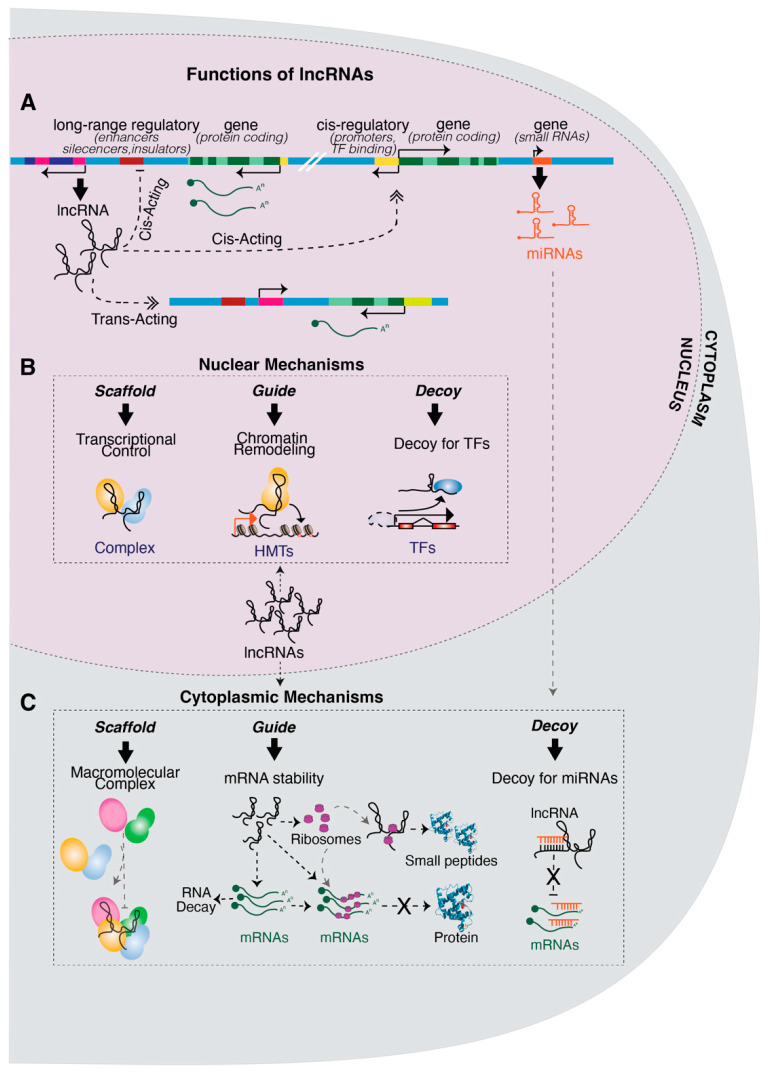
Genomic activity and molecular functions of lncRNAs: (**A**) the panel summarizes the possible modes of action of lncRNA, either in *cis* or in *trans*. The activity in *cis* is exerted on elements in close genomic proximity, while *trans*-acting lncRNAs regulate genes in genomic loci distant from their site of transcription. (**B**) Nuclear lncRNAs can regulate epigenetic and/or gene transcription by acting as: (i) scaffolds for macromolecular complexes; (ii) guides for chromatin-remodeling complexes to specific regulatory regions and (iii) decoys for transcription factors. (**C**) Cytoplasmic lncRNAs can either interact with mRNAs and/or proteins, acting as: (i) scaffolds for promoting the assembly of RNA-binding protein complexes; (ii) guides for modulating mRNA stability, either operating on RNA decay or RNA translation or, alternatively, by being translated in small peptides and associating with proteins and (iii) decoys for miRNAs through MREs.

Among cancers, breast cancer is a life-threatening disease that mirrors the complexity and heterogeneity of the mammary gland. In this context, investigations on lncRNAs have been frequently aimed at the identification of novel or more accurate biomarkers for diagnosis and, above all, the search of novel therapeutic targets that are potentially useful in treating the most-lethal forms of the disease. Importantly, several molecular mechanisms involving lncRNAs have emerged in breast cancer studies and can be considered prototypical for their mode of action. In this review, we aimed at illustrating the complexity of lncRNA mechanisms and their mode of action, focusing on breast cancer as a unifying model system, which is useful for illustrating the biological role and, at the same time, the therapeutic potential of lncRNAs. A comprehensive list of lncRNAs with their mechanisms involved in breast cancer is summarized in Table 1. Below, we will discuss in detail the main mechanisms of lncRNAs, distinguishing those that occur in the nucleus from those that require cytoplasmic localization, as depicted in Figure 1. For each mechanism, we focused on a few lncRNAs with large support from the literature and evidence in breast cancer, which can be used as a prototypical example.

## 2. Functions of lncRNAs in the Nucleus

A large fraction of lncRNAs is expressed almost exclusively in the nucleus [60,61] and, hence, exhibits functions related to nuclear processes, such as the regulation of RNA transcription and RNA splicing or the organization of functionally distinct nuclear domains. Various mechanisms contribute to the nuclear localization of lncRNAs. In general, we can distinguish “passive” mechanisms, favoring the nuclear accumulation of lncRNAs, such as inefficient transcription and low-yield RNA processing (i.e., splicing) [62], from “active” mechanisms based on nuclear retention signals that allow interaction with protein complexes and ribonucleoproteins localized in the nucleus, [63]. Overall, the nuclear functions of lncRNAs are related to the control of gene expression and, hence, fall into two main categories, *cis-* or *trans-acting*, depending on whether the lncRNA influences nearby genes or acts on long-distance regions [64]. Similarly, the activity of lncRNAs can be also categorized as *sequence-dependent* or *-independent*, as it may or may not depend on their exact nucleotide sequence.

### 2.1. lncRNAs as Regulators of Chromatin Status

A recurring theme of nuclear functions is the regulation of the chromatin status. LncRNAs have been shown to influence chromatin organization at different levels. Indeed, the mere act of transcription can modulate the chromatin accessibility of a locus and, thus, lncRNA transcription can function as a *cis*-acting mechanism, influencing the expression of nearby protein-coding genes in a sequence-independent manner [65]. Alternatively, lncRNAs can interact with chromatin modifiers through the recognition of specific binding sites or secondary structures in the lncRNA transcript. Their interaction with proteins can have multiple readouts: lncRNAs can act as a molecular *scaffold*, bridging multiple proteins in a single macromolecular complex, or as a molecular *decoy*, coordinating the regulatory activity in a locus. In both cases, there are examples of *cis-* and *trans*-acting lncRNAs (some of them are reviewed in [66]).

#### HOTAIR

HOX transcript antisense intergenic RNA (*HOTAIR*) is one of the most strikingly cancer-associated lncRNAs [67] and is a typical example of a nuclear lncRNA acting both as a molecular guide and as a scaffold. *HOX* genes are a group of conserved protein-coding genes used in the control of the correct body patterning and are organized into different clusters in the genome. *HOX* genes are tightly regulated during their development and are frequently over-expressed in cancer [68]. The lncRNA *HOTAIR* is a conserved 2.1 kb transcript produced from the *HOXC* locus on chromosome twelve and is composed of six exons, which are actively spliced and polyadenylated [69]. Initially, *HOTAIR* has been suggested to regulate chromatin *in trans* at the distal *HOXD* cluster. Indeed, the knock-down of *HOTAIR* by siRNAs induces a de-repression of the locus and a reduction in the repressive histone modification H3K27me3 [70]. Further studies have suggested that *HOTAIR* acts as a scaffold, coordinating two different chromatin-modifying activities: the deposition of H3K27me3 mediated by Polycomb-repressive complex (PRC2) and the simultaneous demethylation of H3K4me3 by lysine-specific demethylase 1 (LSD1). Two loops in *HOTAIR’s* secondary structure, at the 5′ and 3′ ends, have been proposed to mediate its interaction with PRC2 and LSD1, respectively [70]. This lncRNA is frequently found as dysregulated (mostly over-expressed) in different cancer types. As it relates to breast cancer, *HOTAIR* has been reported to aberrantly target genomic regions other than the *HOXD* cluster, mediating chromatin dysregulation and promoting breast tumor metastasis [8,71].

### 2.2. Enhancer-Like Functions

Enhancers are regions of open chromatins which act as hubs for different transcription-factor-binding sites and operate in a cell-type-specific fashion to activate the expression of target genes (reviewed in [72,73]). Enhancers function on nearby genes (*cis*-acting), which can even be placed at several kb distances thanks to the formation of long-range chromatin interactions. As enhancers are actively transcribed, they also generate non-coding transcripts, which can either be shortly and rapidly degraded (eRNAs) or longer and more frequently processed [74]. These non-coding RNAs may participate in an enhancer function by several types of mechanisms. Here, we describe two lncRNAs involved in breast cancer with their reported enhancer-like functions.

#### CCAT1-L

Colon-cancer-associated transcript-1-long isoform (*CCAT1-L*) is a lncRNA gene, so named as it was found to be highly expressed in colorectal cancer (CRC) samples [75]. *CCAT1-L* is a 5.2 kb long RNA that is enriched at its site of transcription and is chromatin-bound. It is expressed from the 8q24 genomic region, 500 kb upstream of the *myelocytomatosis* (*MYC*) locus. The *CCAT1-L* locus has been associated with several chromatin marks, which are typical of enhancer regions, such as high levels of H3K27Ac and H3K4Me1, low levels of H3K4Me3 and the presence of DNase-I-hypersensitive sites [72,76]. According to these epigenetic marks, the 150 kb long region encompassing *CCAT1-L* has been proposed to act as a putative super-enhancer responsible for controlling MYC expression via a regulatory element embedded in a lncRNA promoter (*MYC*-515) and a downstream regulatory element (*MYC*-335) [77,78]. Three-dimensional conformation capture data have supported the molecular interaction occurring among *MYC-515*, *MYC-335* and MYC promoter. Strikingly, the downregulation of the *CCAT1-L* transcript by antisense oligonucleotides (ASOs) corresponds to a reduction in *MYC* expression and a decreased contact frequency among *MYC*, *MYC*-335 and *MYC*-515. These findings highlight the importance of the lncRNA transcript in mediating enhancer activities, other than DNA features occurring at the locus of its transcription. Overall, the proposed model suggests that the *CCAT1-L* transcript operates in *cis* and participates in establishing long-range contacts that bring the *MYC* locus into proximity with its enhancers thanks to its direct interaction with CCCTC-binding factor (CTCF) [79]. Given the wide oncogenic role of *MYC*, it is not surprising that *CCAT1-L* is frequently expressed at elevated levels in many cancer types. In breast cancer, *CCAT1-L* is a promising prognostic biomarker, as its expression correlates with a decreased overall survival and progression-free survival independently from the receptor status of the disease [80].

#### A-ROD

In some cases, processed (mature) lncRNAs, rather than primary unprocessed transcripts, may directly contribute to enhancer function control. This is the case of *A-ROD,* a lncRNA involved in the regulation of a downstream gene, namely a negative regulator of the Wnt pathway named Dickkopf-1 (*DKK1*) [81]. *A-ROD* is a non-coding transcript originating from a locus acting as an enhancer and is located 130 kb upstream of the *DKK1* locus. In breast cancer cell lines (MCF-7) and samples from breast cancer patients, the two loci showed a correlated expression and were found to be involved in chromatin looping [17]. Interestingly, ASOs targeting the nascent *A-ROD* transcript had no effect on *DKK1* mRNA levels, while siRNAs targeting the mature transcript were able to reduce the expression level of *DKK1* and, at the same time, increase the pausing of RNA polymerase 2 at the *DKK1* transcription start site. The proposed model suggests that *A-ROD* is not involved in chromosomal looping, conversely to *CCAT1-L.* A pre-existing conformation maintains the *A-ROD* locus in proximity to *DKK1,* and the *A-ROD* mature transcript recruits the transcriptional activator EBP4 to enhance *DKK1* transcription. Experiments on the splicing inhibition and transcriptional termination of this lncRNA both supported the fact that the enhancer-like function of *A-ROD* is mediated by the mature transcript. Interestingly, Ntini et al. [17] provided data in support of many other lncRNAs with similar features, as they are transcribed from regions involved in chromatin loops and with a poor association with chromatin, suggesting that the mature form of the lncRNA plays a functional role in gene expression control. In support of this, bioinformatic analyses have proposed that the activity of enhancers is correlated with both the transcription and splicing of their encoded lncRNAs [82,83].

### 2.3. Regulation of Splicing

Recently, lncRNAs were shown to exploit another mechanism of gene expression control by affecting gene splicing. Splicing is a fundamental step in mRNA maturation that allows the excision of introns from transcripts, and the usage of alternative splice sites can affect the production of multiple isoforms subjected to differential regulation in physiology and disease [84]. An emblematic case is the stress-induced lncRNA known as lncRNA associated with SART3 regulation of splicing *(LASTR)*, which is induced by c-JUN together with other survival genes during hypoxia and DNA damage. As c-JUN is frequently overexpressed in epithelial tumors, *LASTR* was found to be highly expressed in most breast cancer subtypes, as reported in The Cancer Genome Atlas—TCGA [21]. This lncRNA is a 714 nt transcript composed of two exons expressed mainly in the nucleus and has been found to interact with SART3, a splicing protein involved in the assembly of the U4/U6 ribonucleic complex, by RNA pulldown assays followed by mass spectrometry [85]. *LASTR* seems to have an impact on splicing control at a global level, as shown by knock-down experiments with ASOs, which resulted in the impairment of the SART3 disassembly from the U4 snRNA and the prevention of the recycling of spliceosome components, increasing intron retention, exon skipping and the non-sense-mediated decay of mRNAs [86].

In normal mammary epithelial cells, the expression of *LASTR* induced by hypoxia facilitates the dissociation of SART3 from the U4/U6 snRNP, preserving cell physiology when stress conditions are present [87]. Similarly, the constitutive overexpression of *LASTR* helps cancer cells to increase their cell fitness by avoiding splicing defects. Strikingly, the knockdown of *LASTR* can sensitize the triple-negative breast cancer cell line MDA-MB-231 to irradiation and impair the tumor growth in mice xenografts, suggesting that this lncRNA is a potential therapeutic target. This work exemplifies how the dynamic regulation of one single lncRNA can largely impact fundamental physiological cellular processes and how cancer cells favorably exploit these simple but effective mechanisms.

### 2.4. Organization of Nuclear Architecture

Some lncRNAs that are abundantly expressed in the nucleus can function in coordinating the organization and activity of functionally distinct nuclear compartments [88]. This is the case of two well-known lncRNAs, named nuclear enriched abundant transcript 1 (*NEAT1*) and metastasis-associated lung adenocarcinoma transcript 1 *(MALAT1)* (also known as *NEAT2*). These lncRNAs are transcribed from proximal loci but are then localized in separate compartments.

*NEAT1* is localized and is essential for the assembly of *paraspeckles*, a dynamic compartment responsible for transcription and RNA processing. *NEAT1* is associated with actively transcribed genes [89]. Thanks to the different protein interacting domains in its sequence, *NEAT1* realizes the precise localization of proteins in this compartment [90]. *NEAT1* expression is dysregulated in many cancer types and is found in the peripheral blood of breast cancer patients [24]. The increased expression of *NEAT1* is associated with a poor prognosis and overall survival [91]. The expression of *NEAT1* is regulated by several tumorigenic transcription factors such as STAT3 or NFkB [92] and contributes to the regulation of the genes responsible for invasion, metastasis and chemoresistance [91].

*MALAT1* is a conserved lncRNA [93] that localizes in *nuclear speckles*, a subnuclear domain where components of the spliceosome concentrate [94]. The current model suggests that *MALAT1* functions in the periphery of nuclear speckles and participates in the positioning of the speckles towards actively transcribed genes, contributing to pre-mRNA splicing [89,95]. Consistent with this model, *MALAT1* knockdown does not impair the formation of nuclear speckles but changes their composition and functionality. The down-modulation of the phosphorylation levels of serine/arginine-rich proteins, which are key splicing components, has been observed upon *MALAT1* silencing, which may explain the impact it has on splicing. *MALAT1* expression has been frequently associated with a poor prognosis, metastasis [26] and chemo- and radio-resistance in several human tumors and in breast cancer [96]. Tandem duplications of the *MALAT1* gene have also been reported [97]. However, Kim et al. provided evidence of a tumor-suppressive role of *MALAT1* in breast cancer cells and primary mammary tumors [25]. According to this work, *MALAT1* interacts with TEAD, preventing the association with the YAP co-activator and the expression of pro-metastatic target genes. These opposite views may not be mutually exclusive but may co-exist in the interpretation of a complex and abundant lncRNA such as *MALAT1*.

## 3. Functions of lncRNAs in the Cytoplasm

A large portion of lncRNAs can exert their functions on the cytoplasm after being transported from the nucleus. A very recent study highlighted that long transcripts that are A/U-rich and with few exons, such as the vast majority of lncRNAs, are dependent on the NXF1 factor for export [98]. Upon arrival, lncRNAs can be sorted into different organelles [99] (i.e., mitochondria or exosomes) or be associated with proteins/nucleic acids. One representative example is the nuclear-encoded lncRNA *SAMMSON*, which is expressed in melanoma cells and is re-located into mitochondria and plays a fundamental role in the regulation of mitochondrial metabolism through the interaction with p32 [57]. How the sorting occurs into different cellular compartments still needs better elucidation; however, it is commonly accepted that it depends on the interaction of lncRNAs with RNA-binding proteins (RBPs) and/or with other RNA species, such as for most (if not all) of the cytosolic functions of lncRNAs. An important caveat for lncRNA/target interaction is represented by the critical stoichiometric ratio between the lncRNA and its target molecule, either a protein or an RNA molecule. In fact, in the case of *cis*-acting lncRNAs, few copies are sufficient to exert a biologically relevant function on a single target gene located in proximity. However, for *trans*-acting lncRNAs, such as all cytoplasmic lncRNAs, it is essential to start with a clear copy number quantification of both the target and the lncRNA in order to verify if a proposed biological mechanism is plausible [64,65]. This especially applies to those lncRNAs acting as competing endogenous RNAs (ceRNAs). This is a mechanism where an lncRNA acts as sponge, soaking up many molecules of a given miRNA and, hence, competing for the interaction of the miRNA with its own set of mRNA targets. A seminal work by Bartel’s group focused on miR-122 as a proof-of-principle in order to challenge the contention that a change in the copy number of a single miRNA target could compete with other shared targets in such a way as to result in a variation in the miRNA’s effects. This work suggests that the miRNA:ceRNA stoichiometry should be better investigated, as the upregulation/downregulation of a single ceRNA may not be sufficient to exert a measurable effect on miRNA activity [100]. Moreover, to further complicate this scenario, lncRNA expression could be associated to a specific tissue (normal vs. cancer) or even restricted to a specific cell type (i.e., rare cell types as stem cells). Thus, to understand the biological effects mediated by the lncRNA, a careful stoichiometric quantification is advisable, accounting for sample purity rather than using a bulk population, which can contain different cell types.

### 3.1. LncRNAs Acting as miRNA Sponges

#### Linc-ROR

LncRNA-regulator of reprogramming, a.k.a., *Linc-ROR*, was initially identified as a competing endogenous RNA (ceRNA) involved in hESC self-renewal. *Linc-ROR* levels are high in hESC and drop down during differentiation [101]. The ceRNA activity was dependent on the microRNA responsive elements (MREs) within *Linc-ROR* sequence and matching with miR-145. This resulted in the sequestering of miR-145 molecules and maintaining the hESC’s pluripotent state by avoiding the miRNA-mediated repression of several embryonic transcription factors (ETFs) [101]. In the breast, *Linc-ROR* was reported to have an impact on the regulation of the epithelial-to-mesenchymal transition (EMT) and the acquisition of chemoresistance and cancer stem cell traits. One of the first pieces of evidence came from a study using MCF10A, a normal breast epithelial cell line, which described *Linc-ROR* as a ceRNA that was able to sponge miR-205, thus causing an increase in ZEB2 levels, a TF-promoting EMT and a well-known target for miR-205. This mechanism can explain the increase in the expression of mesenchymal markers and the effects on proliferation, cell migration and the acquisition of some traits observed upon *Linc-ROR* expression, such as the increased CD44^high^/CD24^low^ population and its capability of forming non-adherent spheroids (mammosphere) [36]. A more recent study on MCF7 cells suggests that *Linc-ROR* acts as ceRNA for miR-194-3, causing an increase in MECP2 levels and, thus, promoting the proliferation, invasion and resistance to rapamycin treatment of breast cancer cells [37]. *Linc-ROR* was also described with another type of mechanism, supporting the estrogen-independent growth of ER+ breast tumors and their resistance to tamoxifen [102]. The proposed mechanism involves the direct inhibition of the phosphatase DUSP7 and the consequent activation of MAPK/ERK signaling, which, in turn, phosphorylates the estrogen receptor and fosters the growth of breast cancer cells and resistance to hormonal chemotherapy [102].

#### H19

The transcript from the *H19* locus was one of the first lncRNAs to be acknowledged. It belongs to a conserved imprinted region located on the human chromosome 11, encoding for a 2.3 kb long cytosolic transcript associated with the epigenetic silencing of the *IGF2* locus [103]. In mouse embryonic and extra-embryonic cell lines, *H19* has also been described as a precursor for miR-675 and capable of regulating placental development by regulating the abundance of *IGF2* at two levels through the imprinting of the gene locus and the silencing of the IGF1R receptor via miR-675 [104].

In breast cancer cells, *H19*/miR-675 expression has been associated with increased proliferation, tumor growth aggressiveness and metastases in vivo. Mechanistically, this was suggested to be dependent on miR-675 activity in b-Cbl and c-Cbl mRNAs, which, in turn, leads to the hyperactivation of EGFR and c-Met and the consequent activation of Akt and Erk signaling [38]. In addition, *H19* has also been described as an miRNA sponge for Let-7, highlighting a prototypical ceRNA mechanism active during muscle differentiation, as shown by Kallen et al. [105]. The ceRNA mechanism was also reported to occur in breast tumors in several reports. For instance, the interaction of *H19* with Let-7c was shown to influence the type of division (symmetric or asymmetric) of cancer stem cells (CSC) by controlling WNT signaling [39]. In another report, *H19* was associated to Let-7a/b and the core pluripotency factor LIN28, a transcription factor critical for stem cells. A positive feedback loop mechanism was described, with H19 competing with LIN28 for Let-7a/b binding, leading to an increase in LIN28 levels, which, in turn, inhibits the generation of mature Let-7a/b molecules from precursors, derepressing all the target genes for Let-7 miRNAs [40]. Lastly, under hypoxic conditions, *H19* was reported to sequester Let-7 miRNAs and to relieve HIF1α mRNA levels. In this report, the *H19*/Let-7/HIF-1α axis was shown to act as metabolic gatekeeper under hypoxia, controlling the switch from OXPHOS to glycolysis [106].

### 3.2. LncRNAs Acting as Guide

#### NORAD

Besides acting as sponges for miRNAs, lncRNAs in the cytosol can also affect mRNA stability, by acting as guide controlling mRNA degradation or mRNA translation. One representative example is the non-coding RNA activated by DNA damage *(NORAD)*. This lncRNA is very abundant in the cytoplasm, where it is bound to PUMILIO1/2 proteins and acts as a decoy. This mechanism depends on the sequence of *NORAD*, which contains several PUMILIO response elements (PRE), a stretch 8 nt long, which is typically located in the in 3′ UTR of PUMILIO target mRNAs. Upon genotoxic stress, *NORAD* acts as reservoir of PUMILIO1/2 proteins and controls genomic stability. In fact, the loss or downregulation of *NORAD* causes a sudden release of PUMILIO1/2 proteins, which bind and accelerate the mRNA turnover of targets involved in DNA repair and DNA replication, thus driving chromosome instability [51].

In colon cancer cells, by combining RNA antisense purification (RAP) and quantitative MS, *NORAD* was identified as a necessary component for the assembly of a ribonucleic complex (*NORAD*-activated ribonucleoprotein complex 1, NARC1) involved in genome stability maintenance. Cells depleted for *NORAD* have increased defects in chromosome segregation, reduced replication fork speed and an altered cell cycle [107].

In breast cancer, *NORAD* was suggested to act as tumor suppressor. A report showed NORAD under transcriptional repression by the YAP/TAZ and NuRD complexes, which usually act as oncogenic factors. In addition, NORAD was shown to act as a decoy for S100P, counteracting its pro-migratory and pro-invasive activity [52].

### 3.3. LncRNA-Encoding Polypeptides

According to their definition, lncRNAs should not have coding functions. However, some studies suggested that small polypeptides can be synthetized from small open-reading frames (ORFs) and can participate in lncRNA-regulatory functions. For instance, in breast cells, *LINC00665* was shown to encode for a micropeptide of 5.5 kDa named CIP2A-BP, which binds CIP2A and competes with the subunit PP2A, an oncogene that promotes tumor progression [31]. Of note is the fact that the translation of LINC00665 is under the control of the TGFβ and SMAD pathways, while the overall levels are not affected. Consistent with this model, the migration and invasion properties of triple-negative breast cancer cells can be inhibited both in vitro and in vivo by the overexpression of the CIP2A-BP protein but not by LINC00665 expression [31].

LncRNA *EPR* (epithelial cell program regulator) was found to be a typical breast epithelial lncRNA, whose expression is inhibited by TGFβ treatment. LncRNA *EPR* was shown to encode for an ~8 kDa small peptide, which localizes at the epithelial cell junctions of mammary glands together with junctional proteins such as ZO-1, CGNL1 and Cortactin [32]. LncRNA *EPR* was suggested to have a dual mechanism. At the RNA level, it can interact with the *Cdkn1a* gene on chromatin and can sustain the expression and stability of *Cdkn1a* mRNA, thus promoting epithelial phenotype and cell cycle arrest [32].

#### PVT1: One lncRNA, Many Functions

Many lncRNAs display multiple regulatory functions that are associated with complex and sometimes conflicting phenotypes. In this category, one representative example is plasmacytoma variant translocation 1 (*PVT1*), a long non-coding RNA. The human *PVT1* gene shows a high level of homology with mouse and rat genomes [108,109].

Six different transcription start sites (TSS) can drive the expression of *PVT1* lncRNA and are distributed in a region of 300 kb and are located downstream of the promoter of the *MYC* oncogene [110]. *MYC* and *PVT1* belong to the 8q24 genomic region, which is frequently altered in cancer. Specifically, this locus is mostly susceptible to amplifications and other structural alterations that lead to the co-amplification of the two genes [111]. Similarly to *MYC*, high expression levels of *PVT1* have been associated with a poor prognosis in breast cancer and other human malignancies [112,113]. The pro-tumorigenic function of *PVT1* can be explained by the activity miRNAs embedded in the lncRNA transcript. *PVT1*, indeed, hosts a miRNA cluster composed of miR-1204, -1205, -1206, -1207-5p and -3p and -1208, which can act as oncomiRs, promoting cell proliferation [114], increasing glycolytic metabolism [115] and suppressing stress-induced apoptosis [116]. However, other studies reported tumor-suppressive functions for the same miRNAs [117,118], suggesting that tissue-specific targets and effects may be involved. In addition, the *PVT1* sequence holds several sites matching miRNAs (MREs), invoking a potential molecular sponge mechanism for the transcript [119]. Indeed, different reports support the possibility that the *PVT1* transcript may sequester miRNAs with tumor-suppressive functions, thus leading to the activation of proliferative and survival pathways [120,121] and the acquisition of metastatic traits.

Moreover, the *PVT1* transcript was shown to interact with the MYC protein *in trans*, regulating MYC stability by interfering with Threonine 58 phosphorylation and proteosome-mediated degradation. MYC, in turn, binds the *PVT1* promoter in two E-boxes sites, creating a positive feedback loop that sustains MYC expression and MYC-induced proliferation [122,123].

In breast cancer, the amplification of the 8q24 region leads to both a gain in the copy number of the *PVT1* gene and the accumulation of genetic alterations at the level of the promoter region of *PVT1*, which abrogates its expression [124]. Starting from this observation, Cho et al. showed that the most-upstream promoter of *PVT1* has a tumor-suppressive function that is independent from the transcription of the lncRNA and aids in tightly regulating the expression of *MYC* [110]. In breast cancer cell lines, the promoters of *PVT1* and *MYC* compete for the binding of intragenic enhancers that are located within the gene body of *PVT1*. When the promoter of *PVT1* is functional, it interacts with the enhancers, which are closer, sustaining the expression of *PVT1* transcript only. When the promoter of *PVT1* is non-functional, the intragenic enhancers rewire it towards the promoter of *MYC* with a topological rearrangement in the 3D genome that boosts the oncogenic expression of *MYC*, thus enhancing cancer cell proliferation [110].

This mechanism of enhancer retargeting seems to be not restricted to the case of the *PVT1-MYC* pair. Oh et al. [125] showed that cancer cells often accumulate mutations in the promoters of genes owing to topological rearrangements of the genome that are able to reinforce the expression of oncogenes. In addition to this, a recent work by Oliviero et al. [126] described an alternative type of relationship occurring between the *Pvt1-Myc* pair in mouse embryonic fibroblasts (MEFs). In this work, a p53-responsive element was found on a downstream TSS for *Pvt1* that, in stress conditions and upon p53 binding, could elicit the expression of the *Pvt1b* isoform and the concurrent repression of *Myc* transcription. The authors suggested that this mechanism is RNA-dependent, as the repression of *Myc* occurs in *cis* in the absence of topological rearrangements and that it is abrogated in the presence of antisense oligonucleotides targeting *Pvt1b* [126]. This mechanism still needs to be clarified in human malignancies, but it highlights a role for lncRNA in adjuvating key stress response pathways such as the one coordinated by p53.

More than 30 years of study have just scratched the complexity of the *PVT1* locus and show the contextual presence of enhancer-like functions of lncRNAs, the *trans*-activity of the *PVT1* transcript in regulating the stability of MYC protein, the contribution of DNA-regulatory elements within a non-coding locus as well as RNA-dependent functions occurring in *cis.* Moreover, this illustrates how cells regulate non-coding RNAs to coordinate multiple physiological or oncogenic activities and realize the fine dosing of key factors.

## 4. Final Remarks

The molecular mechanisms of lncRNAs are manifold, as are their implications in cellular and tumor biology. In this review, we summarized some of the most representative examples that have emerged in recent years and illustrated, in the context of breast cancer, one of the most-studied tumor types. Although it is a common belief that lncRNAs contribute to the key steps of gene expression regulation at either the global level or by optimizing target gene dosage, addressing the complexity of their function or biological activity still represents a major challenge in lncRNA research. Recent technological advances that allow the endogenous investigation of the regulatory function of RNAs together with the development of new sequencing approaches other than short-reading sequencing promise to give new life to this research field and contribute to the development of new fundamental discoveries. In addition to participating in the definition of cancer phenotypes, lncRNAs currently represent promising biomarkers of pathological states and promising therapeutic opportunities. RNA is, by its nature, easier to target and degrade (as compared to proteins or DNA), and the high tissue- and cell-type specificity of lncRNA expression is compatible with highly targeted approaches. All these characteristics make the study of lncRNAs in pathology extremely valuable.

## Figures and Tables

**Table 1:** Long non-coding RNAs and their mechanisms.

Function	Name	Mechanism	Cell Model
Chromatin Regulation	HOTAIR [8]	HOTAIR functions as a scaffold for the repression of the HOX gene cluster. In breast cancer, the increased expression of the transcript causes the re-targeting of PRC2 and boosts invasion and metastasis.	BC cell lines (MDA-MB-231, SK-BR-3, MCF-10A, MCF-7, HCC1954, T47D and MDA-MB-453)
	ANRIL [9,10,11]	ANRIL is transcribed antisense to the INK4/ARF locus containing multiple tumor-suppressing genes responsible for the negative regulation of cell cycle progression. The transcript coordinates in *cis* the epigenetic repression of the locus that is mediated by PRC1 and PRC2. The ANRIL locus bears disease-susceptibility polymorphisms for breast cancer.	Primary samples of breast cancer patients
	LINC02273 [12]	Regulates the activating epigenetic markers H3K4me3 and H3K27ac in the promoter of the AGR2 oncogene, promoting breast cancer metastasis.	Primary samples of metastatic lymph nodes of breast cancer patients and BC cell lines
	CCDC26 [13]	Regulates DNMT1 nuclear localization and DNA methylation. Downregulation of this lncRNA is associated with increased double-strand breaks.	Acute myeloid leukemia
	NBAT-1 [14,15]	Tumor-suppressing lncRNA interacts with PRC2 to realize the epigenetic repression of genes involved in cell proliferation, invasion and migration.	Neuroblastoma and breast cancer
Enhancer-like functions	CCAT1L [16]	CCAT1L establishes a long-range interaction bridging the oncogene MYC with its enhancers.	BC primary samples and cell lines
	A-ROD [17]	The A-ROD mature transcript allows long-range interaction occurring between DKK1 and its upstream enhancer.	MCF-7 and MDA-MB-231
	Eleanor [18,19]	Enables the formation of a TAD that supports the expression of the ESR1 gene and regulates the interaction with a secondary TAD containing the FOXA1 gene, a regulator of apoptosis.	Estrogen receptor positive primary samples and MCF7-LTED (model for long-term estrogen deprivation)
	RAIN [20]	Enhancer-transcribed lncRNA associates with the WDR5 complex, favoring Pol2 transcription at the nearby RUNX2 promoter, leading to the activation of the downstream oncogenic transcription factors and associated features.	Thyroid and breast cancer. Breast cancer cell lines (MCF-7 and MDA-MB-231)
Splicing regulation	LASTR [21]	Stress-induced lncRNA prevents the disassembly and recycling of spliceosome components and globally impairs splicing.	BC cell lines (MDA-MB-468, MDA-MB-231 and MCF10A)
	ZEB2-AS1 [22]	ZEB2-anti pairs with ZEB2 pre-mRNA, inhibiting the efficient splicing of the first exon of the protein-coding gene, promoting the retention of a first intron, which contains an internal ribosome entry site, favoring ZEB2 translation and therefore Ecadherin inhibition and the initiation of EMT.	Breast cancer cell lines MCF-7, BT-20, MDA-MB231 and MDB-MB435
	BC200 [23]	This lncRNA associates through RNA base pairing with the pre mRNA of Bcl-x, interfering with correct splicing and production of the Bcl-xS pro-apoptotic protein.	ER+ breast tumors, MCF-7 and TD47D cell lines
Organization of nuclear architecture	NEAT1 [24,25]	The structural and functional components of paraspecles, a nuclear compartment responsible for RNA processing. A high expression of NEAT1 in breast cancer is associated with increased therapy resistance and stemness.	Breast cancer primary samples and MDA-MB-231
	MALAT1 [26]	MALAT1 is a component of nuclear speckles involved in the splicing of mRNAs. Its overexpression positively correlates with metastasis in breast cancer, however tumor-suppressive roles have been reported for this lncRNA.	Breast cancer primary samples and cell lines
	XIST [27]	XIST is responsible for epigenetic silencing of the inactive X chromosome and its localization at the nuclear periphery. In breast cancer, XIST loss is associated with a dysregulated expression of the mediator complex and favors a less-differentiated, more tumorigenic phenotype.	Breast tumors primary samples, HME and HMLE cell lines
LncRNAs encoding polypeptides	RMRP [28]	Interacts with two RBPs (GRSF1 and HuR) in order to be localized into mitochondria and to regulate oxygen consumption	Hela and HEK293T
	LINC00908 [29]	Generates a small peptide of 60-aa (ASRPS) that binds STAT3, inhibiting its phosphorylation and affecting angiogenesis	BC cell lines
	LINC00961 [30]	Generates a small peptide (SPAR) that interacts with lysosomal v-ATPase to negatively regulate mTORC1 activity	Murine muscle cells
	LINC00665 [31]	Encodes for CIP2A-BP and competes with PP2A for the binding of CIP2A	BT549, MDA-MB-231 and Hs578T
	EPR [32]	Encodes for an 8 kDa protein (EPRp) that localizes and stabilizes epithelial cell junctions	NMuMG and 4T1
	LINC00948 [33]	Generates a micropeptide (MLN) that inhibits SERCA, controlling Ca(2+) uptake into the sarcoplasmic reticulum.	Murine muscle cells
	HOXB-AS3 [34]	Encodes a 53-aa peptide that binds hnRNP A1 that, in turn, regulates pyruvate kinase M splicing and glucose metabolism	Colon cancer cells
Sponges for miRNAs	Lnc-PNUTS [35]	Promotes EMT, migration, invasion in vitro and tumor growth and metastasis in vivo through ZEB1-2 upregulation by sponging miR-205.	HMLE, MCF7, MDA-MB-468, MCF10A and MDA-MB-231
	LincROR [36,37]	Promotes EMT and stem-like state by sponging miR-205 and upregulating ZEB2. Favors BC cells survival in response to rapamycin by sponging miR-194-3p and thus upregulating MECP2.	MCF10A, MDA-MB-231 and MCF7
	H19 [38,39,40]	Acts as an miR-675 precursor and inhibits b- and c-Cbl. Competes with LIN28 for Let7a/b binding, derepressing all Let-7 targets, affecting stem cell self-renewal	BC cell lines
	Lnc-ATB [41,42]	Promotes EMT, migration, invasion and tumor metastasis by sponging the miR-200 family and restoring EMT-TFs as Twist1 and ZEB1-2	Hepatocellular carcinomas and MCF7
	TINCR [43]	Inhibits miR-199-5p transcription by recruiting DNMT1 at its locus. Moreover, TINCR acts as a sponge for miR-199-5p, promoting PD-L1 expression through USP20 stabilization.	BC cell lines
	HOTTIP [44]	Sustains cancer stem-like properties by binding miR-148a-3p and increasing the Wnt signaling pathway	MCF7, T47D and MCF10A
	Lnc-408 [45]	Promotes BC migration and invasion by acting as a sponge for miR-654-5p and upregulating its target LIMK1	BC cell lines
	TMEM105 [46]	Regulates Lactate Dehydrogenase A (LDHA) by sponging miR-1208	MCF7, T47D, MDA-MB-231 and BT549
Post-transcriptional regulation	PDCD4-AS1 [47]	Promotes the stability of PDCD4 mRNA through the formation of the RNA:RNA duplex	MCF10A derivatives (M1-M4)
	NKILA [15,48]	Binds to the NF-κB/IκB complex and inhibits IκB phosphorylation and NF-κB activation, thus blocking BC invasion, tumorigenesis and metastases. Modulates T-cell activation-induced cell death by interacting with the NF-kB/IkB complex, thus regulating the immunotherapy response in breast PDXs.	BC cell lines and BC PDXs
	TINCR [49,50]	Supports mRNA stability thanks to the interaction with the STAU1 RNA-binding protein through 25-nt TINCR-box motifs. It is frequently dysregulated in different human cancer types.	Primary human keratinocytes and cancer cells
	NORAD [51,52]	Acts as decoy of PUMILIO1/2 proteins through its binding at the PUMILIO responsive elements located in PUMILIO 3′ UTR, ultimately controlling genomic stability. In BC, it acts as a decoy of S100P, counteracting its pro-migratory, pro-invasive and pro-metastatic activity.	HCT116, BJ-5Ta cells and BC cell lines
	IRENA [53]	Triggers NF-κB signaling in macrophages through PKR dimerization and increases the production of pro-inflammatory cytokines, ultimately fostering BC chemoresistance.	Breast primary samples, BC cell lines and conditional PyMT-IRENA KO mice
	Lnc-PCIR [54]	Promotes tumorigenesis and metastasis through TAB3 and PABPC4 mRNA/protein up-regulation and TNF-α/NF-κB signaling pathway activation in TNBC.	TNBC cell lines
	LINK-A [55]	Recruits BRK kinase to the EGFR:GPNMB complex, leading to BRK-dependent HIF1α phosphorylation and consequent normoxic HIF1α stabilization, thus promoting BC glycolysis reprogramming	Breast cancer primary samples and MDA-MB-231
	BDNF-AS [56]	Enhancer-transcribed lncRNAs promote tamoxifen resistance by scaffolding the TRIM21-mediated ubiquitination and subsequent degradation of the mTORC inhibitor RNH1.	BC primary samples and BC cell lines (MCF-7, MCF-7R and MDA-MB-231)
Organelles	SAMMSON [57,58]	Interacts with p32 and CARF to enhance mitochondrial metabolism and the synthesis of rRNAs	Melanoma
	GAS5 [59]	Negatively regulates MDH2 acetylation	HEK293T, MDA-MB-231 and MDA-MB-468

## Data Availability

Not applicable.
